# Newcastle Disease Virus Fusion and Haemagglutinin-Neuraminidase Gene Divergence: Implications for Vaccines

**DOI:** 10.3390/vetsci13040368

**Published:** 2026-04-10

**Authors:** Ravendra P. Chauhan, Boguslaw Szewczyk

**Affiliations:** Department of Recombinant Vaccines, Intercollegiate Faculty of Biotechnology, University of Gdansk, ul. Abrahama 58, 80-307 Gdansk, Poland; boguslaw.szewczyk@biotech.ug.edu.pl

**Keywords:** fusion gene, genomic divergence, haemagglutinin-neuraminidase, multivariate analysis, Newcastle disease virus, phylogenetic analysis, vaccine efficacy

## Abstract

Avian orthoavulavirus 1 (AOaV-1), commonly known as Newcastle disease virus (NDV), despite widespread vaccination, remains a significant threat to domestic chickens (*Gallus gallus domesticus*). Currently available live-attenuated NDV vaccines are derived from genotypes I and II lentogenic strains, whereas genetically divergent velogenic strains predominantly caused recent NDV outbreaks. This study examined the extent of genotypic divergence between NDV vaccine strains and field strains using phylogenetic and multivariate analyses of two major antigenic and virulence-associated genes: fusion (F) and haemagglutinin-neuraminidase (HN). A total of 121 full-length NDV-F and 81 NDV-HN gene sequences, representing reported NDV genotypes, were downloaded from GenBank and analysed using maximum-likelihood (ML) phylogenetic trees and principal coordinates analysis (PCoA). The phylogeny revealed genotype-specific clustering for both genes, consistent with current NDV classification. NDV vaccine strains belonging to genotypes I and II formed distinct clades, segregated from the majority of NDV field strains, including velogenic or virulent NDV genotypes. The principal coordinates analysis of both genes further confirmed the phylogenetic clustering of NDV genotypes, indicating increased genomic heterogeneity. These findings suggest genetic segregation of divergent velogenic or virulent genotypes from lentogenic NDV vaccines, requiring biological experiments for determining their efficacy against field strains. This study highlights the importance of molecular surveillance of NDV to monitor its genomic diversity, which is crucial for developing strategies to combat NDV outbreaks in domestic chickens. This study provides an updated, NDV-glycoprotein-gene-based comparative analysis across reported NDV genotypes using phylogenetic and multivariate approaches.

## Simple Summary

Newcastle disease virus (NDV) continues to cause severe outbreaks in poultry despite routine vaccination. The most widely used live vaccines are derived from classical lentogenic (avirulent) strains belonging to genotypes I and II, whereas many recent outbreaks were caused by genetically distinct, virulent NDV genotypes. In this study, we compared the full-length fusion (F) and haemagglutinin-neuraminidase (HN) genes of vaccine strains with representative field strains from all reported NDV genotypes using phylogenetic and multivariate analyses. Our results demonstrate clear genetic divergence between vaccine and circulating strains. These findings highlight the need for continuous molecular surveillance of NDV to monitor its genomic evolution, which is essential to developing strategies to combat the disease in poultry flocks.

## 1. Introduction

Avian orthoavulavirus 1 (AOaV-1), commonly known as Newcastle disease virus (NDV), is a member of the genus *Orthoavulavirus* in the family *Paramyxoviridae* and is the etiological agent of the highly contagious Newcastle disease (ND) in domestic chickens (*Gallus gallus domesticus*) [1,2]. Classified into one serotype—Avian paramyxovirus-1 (APMV-1)—the NDV isolates are grouped into two classes (class I and II) [3]. All class I NDV isolates belong to genotype I, whereas class II isolates are subdivided into genotypes I to XXI, excluding genotype XV, which contains recombinant sequences. NDV forms pleomorphic virions of approximately 200–300 nm diameter, comprising a non-segmented, negative-sense, monopartite RNA genome [4]. Three different genome sizes of NDV have been reported: 15,186 [5], 15,192 [6], and 15,198 nucleotides [7], reflecting progressive evolutionary diversification driven by error-prone RNA polymerase activity.

The NDV genome has two glycoprotein genes: fusion (F) and haemagglutinin-neuraminidase (HN), the determinants of virulence and antigenicity, respectively [8]. The amino acid sequence at the fusion protein cleavage site (F0) is a major determinant of the pathogenicity of NDV strains. The lentogenic NDV strains have a mono- or dibasic amino acid sequence ‘^112^(E/Q)-(G/E)-R-Q-(G/E)-R↓L^117^’ at the F0 cleavage site, which is cleaved only by blood clotting factor Xa-like proteases, is limited to the respiratory and enteric tracts, and therefore tends to cause limited disease. The velogenic NDV strains contain polybasic residues at the F0 cleavage site, specifically ‘^112^R(K)RQR(K)R^↓^F^117^’, which are the preferred recognition sites for furin-like proteases typically present in most cells [9]. This facilitates systemic infection, thereby increasing disease severity.

Classified into 21 genotypes, NDV infections harbour quasispecies [2,8]. Based on the amino acid sequence of the F0 cleavage site and a clinical weighted score—intracerebral pathogenicity index (ICPI)—ranging from 0.0 to 2.0, NDV strains are categorised into three pathotypes: lentogenic, mesogenic, and velogenic [10]. NDV strains with ICPI ≥0.7 are classified as virulent; mesogenic (ICPI = 0.7 to 1.4) and velogenic (ICPI = 1.5 to 2.0) strains cause ND outbreaks in commercial poultry [10].

Clinical signs of NDV disease in chickens may vary by NDV genotype, including respiratory illness, greenish diarrhoea, weight loss, decreased egg production, haemorrhagic and ulcerative lesions in the gastrointestinal tract, depression, and mortality [11,12]. The velogenic NDV strains can cause up to 100% mortality in susceptible chickens within one week [12]. As of March 2026, active NDV outbreaks have been reported to the World Organisation for Animal Health (WOAH) from Poland, Germany, Spain, Israel, Peru, Liberia, and Malaysia. During 2025–2026, 19 countries from Asia, Africa, Europe, and the Americas reported NDV outbreaks to the WOAH [13].

Vaccination of domestic chickens is the only protection against ND-related mortality. The lentogenic live vaccines, such as LaSota and Hitchner-B1 (also known as B1; genotype II), as well as V4 and I-2 (genotype I), are among the most commonly used vaccines to protect commercial poultry against ND outbreaks [14]. The live vaccines, such as Roakin (genotype II) and Mukteswar (genotype III) [15], due to increased virulence and potential to cause mortality in young, vaccinated chickens, have been banned in many countries [14], making LaSota and B1 the most widely used lentogenic NDV vaccines to date. However, these vaccines confer limited protection against morbidity and mortality and are inefficient in preventing virus shedding in immunised chickens [14]. This has led to the circulation of virulent NDV strains among vaccinated chickens in many countries [11,16,17].

To date, no comprehensive comparative analysis of full-length NDV-F and -HN gene divergence encompassing all currently recognised NDV genotypes has been performed using an integrated approach combining maximum-likelihood (ML) phylogenetics and principal coordinates analysis (PCoA). Therefore, this study examines the genotypic divergence of the NDV-F and -HN genes relative to vaccine strains and discusses its implications for the efficacy of currently deployed NDV vaccines. This study uses selected nucleotide sequences of the NDV-F and -HN genes, representing all reported NDV genotypes, to provide an updated, comparative analysis using phylogenetic and multivariate analyses.

## 2. Materials and Methods

### 2.1. NDV-F and -HN Gene Sequence Selection Criteria

The full-length gene sequences of NDV-F (*n* = 121) and -HN (*n* = 81), representing all NDV genotypes except genotype XV, which is a recombinant genotype, were downloaded from GenBank in November 2025. Sequence selection was performed using a stratified representative sampling approach to ensure comprehensive coverage of NDV genetic diversity. Selection criteria were defined as follows: (i) only full-length coding sequences of the F and HN genes were included; (ii) sequences with clearly assigned genotypes based on GenBank annotation and/or published literature were included; and (iii) multiple representative sequences were retained for each genotype to capture intra-genotypic diversity. To minimise redundancy in the dataset, sequence-similarity filtering was performed following multiple sequence alignment. Pairwise nucleotide identity was calculated from the aligned sequences using MEGA v11.0.13 [18]. Sequences sharing ≥99.5% nucleotide identity and originating from the same outbreak, geographical location, and year of isolation were considered redundant; in such cases, a single representative sequence was retained. In cases where highly similar sequences differed in host species, geographical origin, or sampling time, they were retained to preserve epidemiological and evolutionary diversity. The final dataset was curated to ensure broad representation across geographical regions, host species, and temporal distribution (1940s–2023), as well as inclusion of different NDV pathotypes (lentogenic, mesogenic, and velogenic/virulent). Details of all analysed sequences, including accession numbers, host, country, year of isolation, genotype, and pathotype, are provided in Supplementary Table S1.

### 2.2. Phylogenetic and Multivariate Analyses

NDV-F and -HN gene sequences were aligned using the MUSCLE algorithm in MEGA v11.0.13. The best-fit nucleotide substitution model for each dataset was determined using the “Find Best DNA/Protein Models” function in MEGA v11.0.13 based on the Bayesian Information Criterion (BIC). The General Time Reversible model with gamma-distributed rate variation and a proportion of invariant sites (GTR + G + I) was identified as the optimal model and subsequently applied for phylogenetic reconstruction. Maximum-likelihood (ML) phylogenetic trees were constructed in MEGA v11.0.13 using the GTR + G + I model, with 1000 bootstrap replicates to assess node support. Bootstrap values ≥60% were considered indicative of moderate to strong support for inferred clades. Gaps were handled using the partial deletion option with a 95% site coverage cutoff.

For multivariate analysis, distance matrices were generated from MAFFT (v7.490)-aligned sequences in Geneious Prime v2025.1.2 (Biomatters) using pairwise nucleotide distances. The resulting distance matrices were exported in .csv format and subsequently converted to .xml format in Microsoft Excel v2510 to ensure compatibility with PAST (Paleontological Statistics) v4.02, which requires structured matrix input for ordination analyses. Principal coordinates analysis (PCoA) was performed in PAST using the distance matrix as input, with eigenvalue decomposition used to project the samples into a reduced-dimensional space. No additional data transformation or scaling was applied beyond the default settings of the PAST v4.02 software.

### 2.3. Nucleotide and Amino Acid Sequence Analyses

To evaluate genetic divergence, nucleotide and amino acid percent identities between the LaSota vaccine and field genotypes were determined using pairwise alignments in Geneious Prime v2025.1.2 (Biomatters). Furthermore, amino acid substitutions in the NDV-F and -HN proteins were identified through multiple sequence alignments in MEGA v11.0.13, using the LaSota strain as the reference and representative field strains.

## 3. Results and Discussion

**Figure 1:**
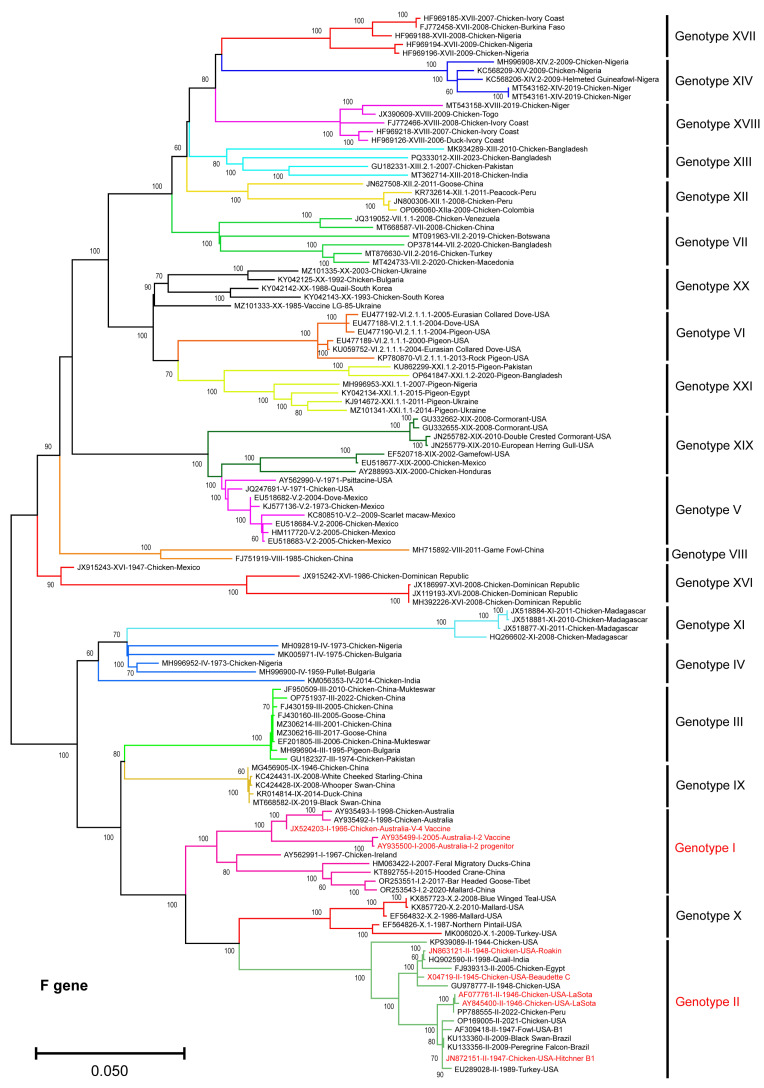
Phylogenetic analysis of the NDV-F gene sequences. The ML phylogenetic tree constructed using full-length F gene nucleotide sequences (*n* = 121) clustered according to the NDV genotypes, with vaccine strains belonging to genotypes I and II highlighted in red. The bootstrap values (>60%) were shown at the nodes. The scale bar indicates nucleotide substitutions per site.

The ML phylogenetic tree (Figure 1), constructed from full-length nucleotide sequences (*n* = 121) of the NDV-F gene, representing isolates spanning all reported NDV genotypes, revealed well-supported genotype-level clustering, with individual genotypes forming distinct monophyletic groups. This observation was consistent with established NDV classification [2]. The genotype I and II strains, which include currently used live-attenuated vaccine strains, clustered separately from the majority of the NDV isolates. The genotype I sequences formed a compact clade with relatively short branch lengths, indicative of limited intra-genotype genetic divergence. Similarly, genotype II strains clustered together, including the reference vaccine strains LaSota and Hitchner-B1, and showed greater genetic divergence from the virulent field strains belonging to genotypes V, VI, VII, XIII, XIV, XVII, and XVIII. Notably, genotype VII and several other broadly circulating genotypes formed highly divergent clades, indicating substantial nucleotide divergence relative to vaccine-related genotypes I and II.

**Figure 2:**
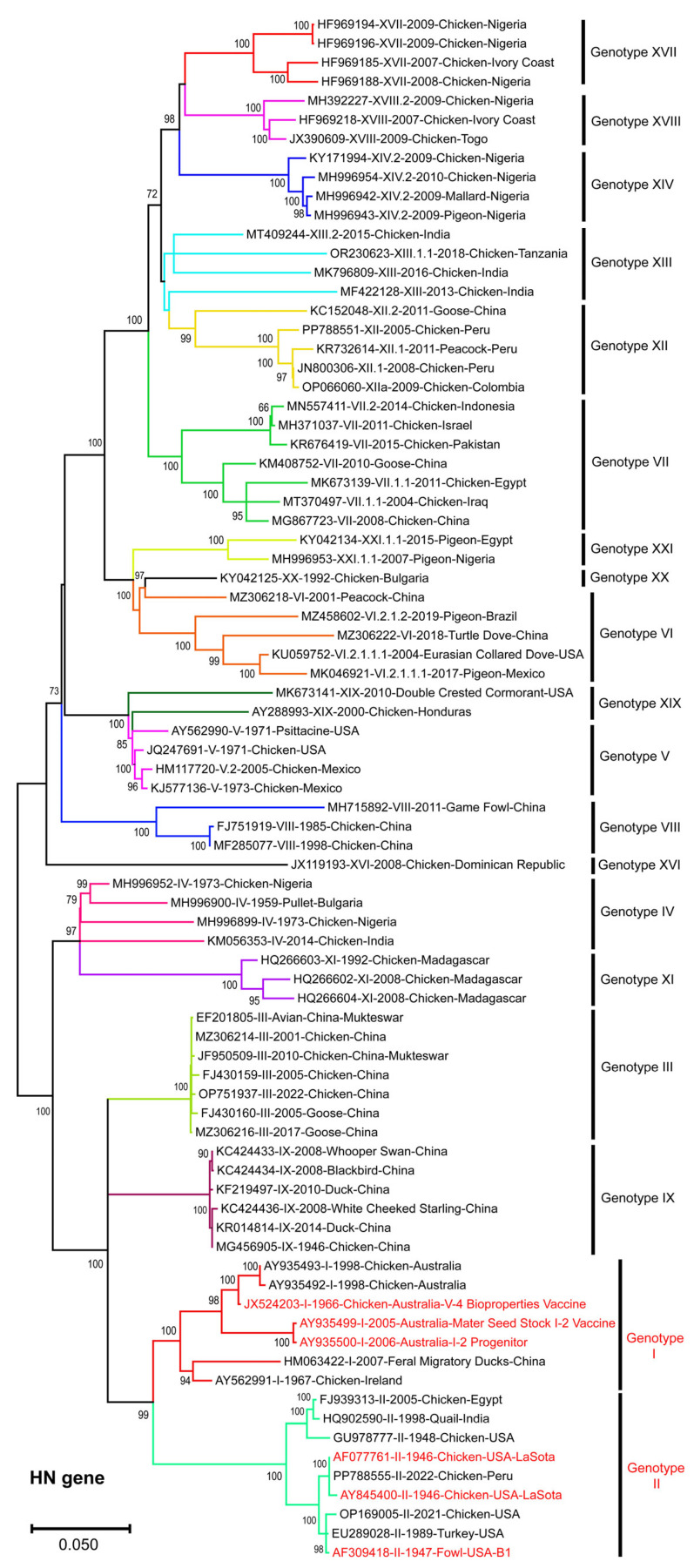
Phylogenetic analysis of NDV-HN gene sequences. The ML tree constructed using full-length HN gene sequences (*n* = 81), representing all NDV genotypes, clustered into well-supported, genotype-specific clades. The vaccine strains belonging to genotypes I and II are highlighted in red. The bootstrap values (>60%) are shown at the nodes. The scale bar represents nucleotide substitutions per site.

Similarly, the ML phylogenetic tree based on full-length NDV-HN gene sequences (*n* = 81) also showed genotype-specific clustering among NDV isolates (Figure 2). It formed monophyletic clades, with the majority of field NDV genotypes diverging from vaccine-related strains. Similar to F-gene-based phylogeny, the HN-gene-based ML tree formed distinct clades, highlighting evolutionary divergence from NDV vaccine strains.

**Figure 3:**
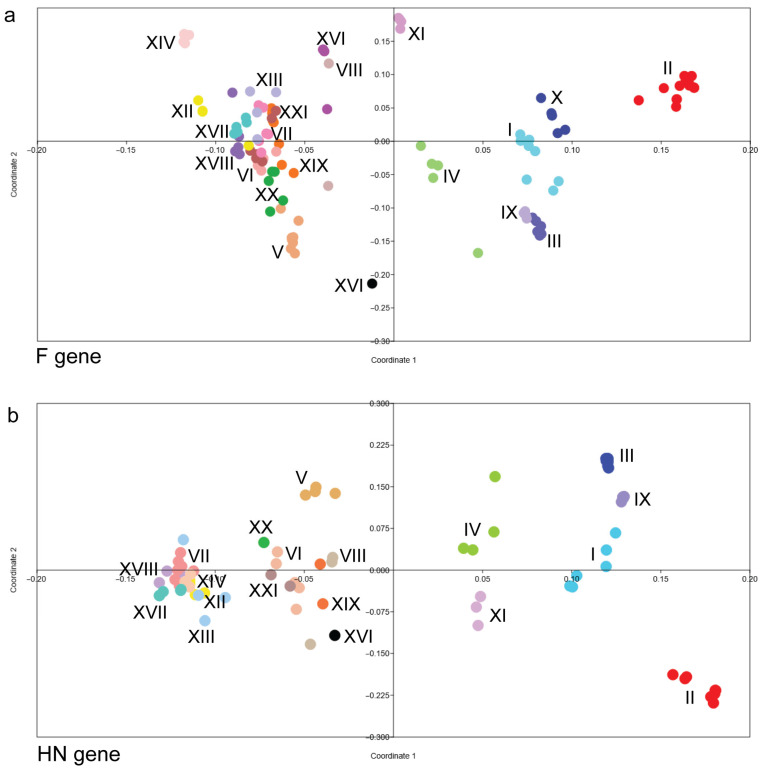
Principal Coordinates Analysis (PCoA) of NDV genotypes based on glycoprotein gene sequences. The genetic distance and clustering of NDV genotypes were visualised using PCoA based on the (**a**) F gene and (**b**) HN gene sequences. Each coloured dot represents an individual viral isolate, while Roman numerals (I–XXI) denote specific NDV genotypes. Isolates belonging to the same genotype consistently cluster together, reflecting high intra-genotypic sequence homology. Genotype II (red) shows significant divergence from the other genotype clusters.

To further examine genetic relationships among NDV genotypes, PCoA was performed using pairwise genetic distances derived from F gene sequences (Figure 3a). While NDV genotype I and II strains formed compact clusters with minimal overlap with other NDV genotypes, field NDV genotypes exhibited broader, more dispersed distributions across the coordinate space, reflecting greater genetic heterogeneity. The PCoA based on pairwise genetic distances derived from HN gene sequences (Figure 3b) further supported the phylogenetic clustering of the NDV genotypes.

Overall, this study, based on phylogenetic and multivariate analyses of selected full-length NDV-F and -HN gene sequences, revealed genotype-specific clustering, highlighting genomic divergence between vaccine strains and field isolates of NDV. The compact clustering and shorter branch lengths observed for genotype I and II strains in the ML phylogenetic tree likely reflect their historical use as vaccine strains and relatively limited evolutionary diversification compared to the field strains. In contrast, the extended branch lengths and broader dispersion of circulating genotypes, such as VII and XIII, among others, suggest ongoing evolution under field conditions.

The spatial separation of vaccine-related genotypes I and II from circulating field genotypes in multivariate space reinforces the phylogenetic findings and provides a quantitative representation of accumulated nucleotide divergence. The minimal overlap between genotype I and II vaccine strains and several velogenic genotypes underscores the significant genomic divergence between classical NDV vaccine strains and circulating NDV field genotypes, emphasising the importance of continued molecular surveillance and antigenic characterisation for updated NDV vaccines.

Percent pairwise identities in nucleotide and amino acid sequences (Table 1) further supported the phylogenetic and multivariate clustering of NDV genotypes. Analysis of the percent pairwise similarities reveals significant genetic divergence between the LaSota vaccine strain and various NDV field isolates. While the vaccine strain exhibits near-identity with genotype II (98.9–99.3% similarity), it shows a marked decrease in homology compared to most field genotypes. Notably, genotypes XIV, XVI, and XVII consistently display nucleotide similarities below 84% for both the F and HN genes, suggesting continuous evolution of field strains circulating globally.

The proteolytic cleavage site (F0) of the fusion protein is a primary determinant of NDV virulence, and the motifs identified in this study align with established NDV pathotyping criteria. The vaccine strains, LaSota and B1, harboured the characteristic lentogenic motif ^112^GRQGRL^117^, defined by a lack of multiple basic amino acids and the presence of leucine at position 117. Conversely, the majority of field genotypes (III–XXI) exhibited multibasic motifs at the F0 cleavage site, most commonly RRQKRF or RRQRRF, with a phenylalanine at position 117, associated with systemic replication and high virulence (Table 2). Notably, the polybasic sites in genotype XI (RRRRRF) and the lack of multibasic sites in genotype X motif highlight the ongoing evolutionary dynamics at this critical genomic region, which remains a vital target for molecular surveillance and virulence assessment.

**Figure 4:**
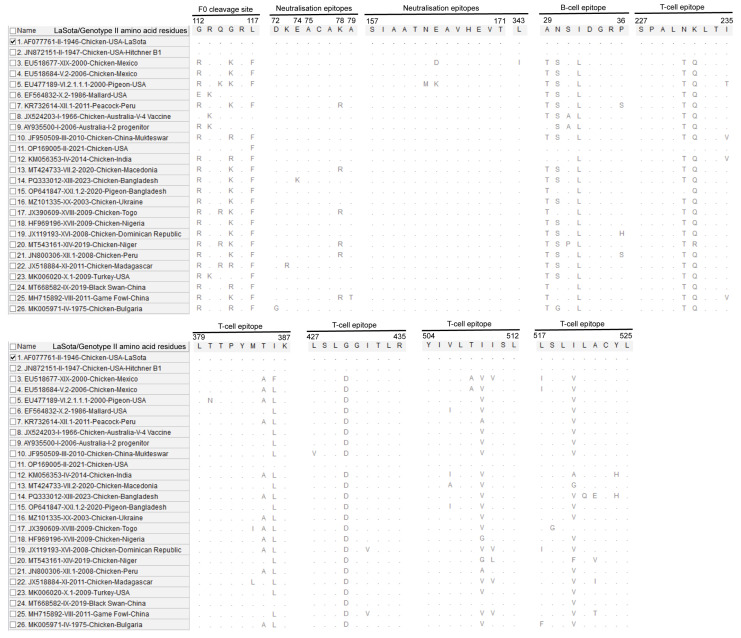
A comparative amino acid alignment of the NDV-F protein functional and immunogenic domains illustrates sequence divergence between the LaSota vaccine strain and representative field genotypes. Dots represent identity with the reference sequence, while letters indicate specific substitutions within key functional regions. Amino acid substitutions within neutralisation, B-cell, and T-cell epitopes highlight antigenic drift that may impact vaccine-mediated protection against genotypically diverse field strains. The analysed antigenic and neutralisation epitopes have been previously characterised [19,20]. The mark represents the reference strain.

A comparative analysis of NDV-F amino acid sequences reveals substantial divergence between the LaSota vaccine strain and diverse field genotypes across critical immunogenic and functional domains (Figure 4). The antigenic and neutralisation epitopes have been characterised in previous studies [19,20]. Beyond the well-characterised multibasic F0 cleavage site in virulent genotypes, notable substitutions were observed within primary neutralisation epitopes, B-cell, and T-cell epitopes, suggesting antigenic diversity across NDV isolates. The sequence variations at key residues likely contribute to the observed antigenic mismatch between the genotype II-based LaSota vaccine and circulating field strains.

**Figure 5:**
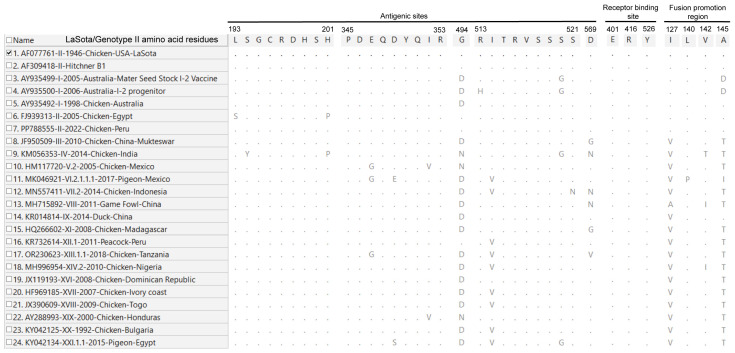
Amino acid sequence alignment of the NDV-HN protein globular head domain across representative genotypes. The alignment compares field isolates (genotypes I–XXI) against the LaSota (genotype II) vaccine strain. Dots represent identity with the reference sequence, while letters indicate specific substitutions within key functional regions. While the receptor binding sites (E401, R416, and Y526) remain strictly conserved across all strains, significant divergence is observed in the antigenic sites, particularly at positions 494, 514, and 569. Additionally, nearly all field genotypes (III–XXI) exhibit consistent substitutions in the fusion-promotion region (e.g., I127V and A145T). The mark represents the reference strain.

Analysis of the HN protein globular head domain reveals extensive amino acid substitutions in key antigenic sites and the fusion-promotion region compared to the LaSota vaccine strain (Figure 5). While the receptor-binding sites (E401, R416, and Y526) remained entirely conserved, significant drift was observed in major antigenic epitopes, particularly at positions 494, 514, and 569. Furthermore, amino acid substitutions, including I127V and A145T within the fusion-promotion region of field genotypes (III–XXI), likely suggest a shift in the dynamics of HN-F protein interactions compared to those of traditional vaccine strains. These cumulative mutations may likely contribute to the antigenic mismatch between the LaSota vaccine and circulating field viruses, potentially leading to increased viral shedding and reduced cross-protection in vaccinated populations. Further biological characterisation will inform the impact of these amino acid substitutions on the efficacy of the LaSota vaccine. The antigenic sites, receptor-binding sites, and fusion-promotion region analysed in this study have been functionally characterised previously [21,22,23].

There is significant divergence in the NDV-F and -HN genes between vaccine-related genotypes I and II and currently circulating field strains, such as genotypes VII, XIII, XIV, and XVII, among others. While genotype mismatch does not always compromise protection from mortality, it might reduce the vaccine’s efficacy in cross-protection against replication and shedding of velogenic strains, enabling continued circulation and transmission in vaccinated flocks [24,25]. While mutations in the F gene may influence fusion [26], substitutions in the HN gene in or around neutralising epitopes may reduce cross-protection [27], contributing to incomplete vaccine-mediated immunity, as observed in challenge and field studies [28]. Genotype-matched or recombinant experimental NDV vaccines, such as genotype VII.1.1, have been reported to be more efficacious at protecting against and reducing virus shedding than non-genotype-matched or classical LaSota vaccines [24,29]. Although classical lentogenic vaccines induce robust systemic humoral responses, they fail to provide adequate mucosal immunity, resulting in continued virus shedding and transmission [25].

At the molecular level, the antigenic mismatch between classical vaccine strains (e.g., LaSota and B1; genotype II) and velogenic field strains is caused by amino acid substitutions within key neutralising epitopes of both the F and HN glycoproteins [30]. The F protein, particularly the F1 subunit, contains major neutralising epitopes and heptad repeat regions essential for membrane fusion [31]. Amino acid substitutions within these regions could alter epitope conformation and reduce antibody binding affinity, thereby diminishing neutralisation efficiency. Furthermore, variations at the F protein cleavage site may influence virulence and tissue tropism, affecting viral replication and antigen presentation in vivo [32].

The HN protein plays a dual role in receptor binding and neuraminidase activity and is a major target of neutralising antibodies [33]. Amino acid substitutions within the globular head domain of HN, particularly in regions involved in receptor recognition and antibody binding, can modify antigenicity without necessarily affecting receptor-binding function [34]. Such substitutions may cause immune escape from vaccine-induced antibodies. Structural modelling and epitope mapping studies have reported that certain HN substitutions located within or adjacent to neutralising epitopes may introduce antigenic drift, thereby reducing cross-neutralisation efficiency against heterologous genotypes [15,35]. Due to antigenic drift, virus shedding may persist in chickens, as evidenced by challenge studies using heterologous velogenic NDV strains, such as genotypes VII [30] or IX, despite acceptable serological titers induced by the genotype II-based LaSota vaccine [36]. The persistent residual shedding is epidemiologically significant, as it facilitates horizontal transmission and circulation.

NDV vaccines targeting conserved epitopes of F and HN proteins would exert broadly cross-protective immune responses that are least affected by genotype-specific antigenic drift [37]. Experimental studies using epitope-based, recombinant, and viral vector-based NDV vaccine platforms have demonstrated enhanced heterologous protection and reduced virus shedding against divergent NDV genotypes. These findings support the concept that conserved-epitope-based vaccination may be a promising strategy for enhanced protection against NDV outbreaks [38]. Furthermore, advances in immunoinformatics have enabled the rational design of multi-epitope constructs that maintain conformational integrity while expanding antigenic coverage [20].

Studies have shown that vaccines matched to currently circulating genotypes, such as NDV genotypes VII or XII, provide better protection than the genotype II-based LaSota vaccine [39,40,41]. Challenge studies have reported enhanced efficacy of genotype-matched vaccines exhibiting reduced virus shedding and improved protection. Virus-like particle (VLP)-based NDV vaccines expressing genotype-relevant F [42] and HN proteins [43], as well as vectored vaccines using the herpesvirus of turkey (HVT) platform [44], have shown promise in inducing durable immunity.

This study has certain limitations, including reliance on publicly available data, which might introduce uneven geographical representation. Also, the genetic divergence was assessed at the nucleotide level without direct antigenic characterisation or structural modelling of epitope regions.

Continued surveillance of circulating genotypes, coupled with antigenic characterisation, will be essential to align vaccination strategies with the evolving NDV disease burden in poultry flocks. Given the global predominance of genotype VII and other divergent genotypes in many endemic regions, continued reliance on genotype II-based vaccines may sustain a scenario in which mortality is controlled but viral transmission persists. The incomplete suppression of viral replication in vaccinated poultry flocks may facilitate silent circulation and periodic NDV outbreaks. Future research integrating epitope analysis, antigenic mapping, and challenge studies will help clarify the effects of genetic divergence and support the design of more efficacious NDV vaccines.

## Figures and Tables

**Table 1:** Percent pairwise similarities in nucleotide and amino acid sequences between LaSota vaccine and field strains.

Genotypes (Accession Number/Year/Host/Country)	NDV-F Percent Similarity (%)		NDV-HN Percent Similarity (%)	
Reference strain: LaSota vaccine (AF077761)	Nucleotide	Amino acid	Nucleotide	Amino acid
I: HM063422-2007-Feral_migratory_ducks-China	89.0	92.8	88.7	94.3
II: EU289028-1989-Turkey-USA	98.9	99.3	99.0	99.1
III: FJ430159-2005-Chicken-China	88.5	90.1	87.9	89.8
IV: KM056353-2014-Chicken-India	86.8	90.4	85.8	89.8
V: AY562990-1971-Psittacine-USA	86.0	88.4	85.7	90.2
VI: KU059752-2004-Eurasian collared dove-USA	85.0	88.6	83.0	88.6
VII: JQ319052-2008-Chicken-Venezuela/F VII: MN557411-2014-Chicken-Indonesia/HN	84.5	88.6	82.1	87.9
VIII: MH715892-2011-Game_fowl-China	84.2	87.9	81.7	87.9
IX: MT668582-2019-Black_swan-China/F IX: KR014814-2014-Duck-China/HN	89.3	92.2	88.5	91.8
X: EF564826-1987-Northern_pintail-USA/F	90.3	93.9	NA	NA
XI: JX518881-2010-Chicken-Madagascar/F XI: HQ266602-2008-Chicken-Madagascar/HN	83.2	87.0	83.3	87.9
XII: JN627508-2011-Goose-China/F XII: KC152048-2011-Goose-China/HN	84.1	88.8	82.2	86.2
XIII: MT362714-2018-Chicken-India/F XIII: MT409244-2015-Chicken-India/HN	83.9	88.4	82.6	87.6
XIV: MT543162-2019-Chicken-Niger/F XIV: KY171994-2009-Chicken-Nigeria/HN	81.3	86.3	82.5	87.4
XVI: JX119193-2008-Chicken-Dominican_Republic	83.3	86.8	81.9	86.7
XVII: HF969185-2007-Chicken-Ivory_Coast	83.6	87.0	81.2	87.9
XVIII: JX390609-2009-Chicken-Togo	84.2	88.6	81.8	87.7
XIX: AY288993-2000-Chicken-Honduras	84.7	88.8	83.0	88.6
XX: KY042125-1992-Chicken-Bulgaria	84.7	89.5	83.7	89.7
XXI: KY042134-2015-Pigeon-Egypt	83.8	88.6	83.2	88.8

NA = not available.

**Table 2:** Comparison of F0 cleavage site motifs (amino acid positions 112–117) among NDV vaccine strains and field genotypes.

Strain/Genotype	Cleavage Site Motif (112–117)	Pathotype
Vaccine strain: I-2 progenitor/I	RKQGRL	Lentogenic
Vaccine strain: V4/I	GKQGRL	Lentogenic
Vaccine strain: LaSota/II	GRQGRL	Lentogenic
Vaccine strain: Hitchner-B1/II	GRQGRL	Lentogenic
Beaudette/II	RRQKRF	Virulent
III/IV/IX	RRQRRF	Mesogenic/Virulent
V/VIII/XII/XVI/XVII/XXI	RRQKRF	Virulent
VI	RRKKRF	Virulent
VII/XIII/XIV/XVIII/XX	RRQKRF/RRRKRF	Virulent
X	EKQGRL/ RKQGRF	Lentogenic
XI	RRRRRF	Virulent
XIX	KRQKRF	Virulent

## Data Availability

The data presented in this study are openly available in NCBI-GenBank at https://www.ncbi.nlm.nih.gov/nucleotide/ (accessed on 10 November 2025).

## Supplementary Materials

The following supporting information can be downloaded at: https://www.mdpi.com/article/10.3390/vetsci13040368/s1. Supplementary Table S1. Newcastle disease virus (NDV) fusion (F) and haemagglutinin-neuraminidase (HN) genes, analysed in the study.

## Abbreviations

The following abbreviations are used in this manuscript:

AOaV-1	Avian orthoavulavirus 1
NDV	Newcastle Disease Virus
F	Fusion
HN	Haemagglutinin-Neuraminidase
PCoA	Principal Coordinates Analysis
PAST	PAleontological STatistics

## References

[1] King A.M.Q., Adams M.J., Carstens E.B., Lefkowitz E.J. (2012). Virus Taxonomy: Ninth Report of the International Committee on Taxonomy of Viruses.

[2] Dimitrov K.M., Abolnik C., Afonso C.L., Albina E., Bahl J., Berg M., Briand F.X., Brown I.H., Choi K.S., Chvala I. (2019). Updated unified phylogenetic classification system and revised nomenclature for Newcastle disease virus. Infect. Genet. Evol..

[3] Ballagi-Pordány A., Wehmann E., Herczeg J., Belák S., Lomniczi B. (1996). Identification and grouping of Newcastle disease virus strains by restriction site analysis of a region from the F gene. Arch. Virol..

[4] Ganar K., Das M., Sinha S., Kumar S. (2014). Newcastle disease virus: Current status and our understanding. Virus Res..

[5] de Leeuw O., Peeters B. (1999). Complete nucleotide sequence of Newcastle disease virus: Evidence for the existence of a new genus within the subfamily Paramyxovirinae. J. Gen. Virol..

[6] Absalón A.E., Mariano-Matías A., Vásquez-Márquez A., Morales-Garzón A., Cortés-Espinosa D.V., Ortega-García R., Lucio-Decanini E. (2012). Complete genome sequence of a velogenic Newcastle disease virus isolated in Mexico. Virus Genes.

[7] Czeglédi A., Ujvári D., Somogyi E., Wehmann E., Werner O., Lomniczi B. (2006). Third genome size category of avian paramyxovirus serotype 1 (Newcastle disease virus) and evolutionary implications. Virus Res..

[8] Jadhav A., Zhao L., Liu W., Ding C., Nair V., Ramos-Onsins S.E., Ferretti L. (2020). Genomic Diversity and Evolution of Quasispecies in Newcastle Disease Virus Infections. Viruses.

[9] de Leeuw O.S., Koch G., Hartog L., Ravenshorst N., Peeters B.P.H. (2005). Virulence of Newcastle disease virus is determined by the cleavage site of the fusion protein and by both the stem region and globular head of the haemagglutinin-neuraminidase protein. J. Gen. Virol..

[10] WOAH (2021). Newcastle Disease (Infection with Newcastle Disease Virus)—OIE Terrestrial Manual 2021. https://www.woah.org/en/disease/newcastle-disease/.

[11] Umali D.V., Ito H., Suzuki T., Shirota K., Katoh H., Ito T. (2013). Molecular epidemiology of Newcastle disease virus isolates from vaccinated commercial poultry farms in non-epidemic areas of Japan. Virol. J..

[12] Igwe A.O., Ismaila S., Okoye J.O.A. (2018). Response of cyclophosphamide-treated broiler chickens to challenge with velogenic Newcastle disease virus. J. Appl. Anim. Res..

[13] WOAH (2026). Events Management.

[14] Hu Z., He X., Deng J., Hu J., Liu X. (2022). Current situation and future direction of Newcastle disease vaccines. Vet. Res..

[15] Lu X., Liu X., Song Q., Wang X., Hu S., Liu X. (2022). Amino Acid Mutations in Hemagglutinin-Neuraminidase Enhance the Virulence and Pathogenicity of the Genotype III Newcastle Disease Vaccine Strain After Intravenous Inoculation. Front. Vet. Sci..

[16] Mariappan A.K., Munusamy P., Kumar D., Latheef S.K., Singh S.D., Singh R., Dhama K. (2018). Pathological and molecular investigation of velogenic viscerotropic Newcastle disease outbreak in a vaccinated chicken flocks. Virusdisease.

[17] Yadeta W., Amosun E., Mohammed H., Woldemedhin W., Sherefa K., Legesse A., Deresse G., Birhanu K., Abayneh T., Getachew B. (2024). Isolation and Genetic Characterization of Genotype VII Velogenic Pathotype Newcastle Disease Virus from Commercial Chicken Farms in Central Ethiopia, Distinct from the Local Vaccine Strains. Viruses.

[18] Tamura K., Stecher G., Kumar S. (2021). MEGA11: Molecular Evolutionary Genetics Analysis Version 11. Mol. Biol. Evol..

[19] Toyoda T., Gotoh B., Sakaguchi T., Kida H., Nagai Y. (1988). Identification of amino acids relevant to three antigenic determinants on the fusion protein of Newcastle disease virus that are involved in fusion inhibition and neutralization. J. Virol..

[20] Randriamamisolonirina N.T., Razafindrafara M.S., Maminiaina O.F. (2025). Design of a Multi-Epitope Vaccine against the Glycoproteins of Newcastle Disease Virus by Using an Immunoinformatics Approach. ACS Omega.

[21] Tavassoli A., Soleymani S., Housaindokht M.R. (2024). Nucleotide sequence characterization, amino acid variations and 3D structural analysis of HN protein of the NDV VIId genotype. Vet. Med. Sci..

[22] Khattar S.K., Yan Y., Panda A., Collins P.L., Samal S.K. (2009). A Y526Q mutation in the Newcastle disease virus HN protein reduces its functional activities and attenuates virus replication and pathogenicity. J. Virol..

[23] Omony J.B., Wanyana A., Mugimba K.K., Kirunda H., Nakavuma J.L., Otim-Onapa M., Byarugaba D.K. (2016). Disparate thermostability profiles and HN gene domains of field isolates of Newcastle disease virus from live bird markets and waterfowl in Uganda. Virol. J..

[24] Sultan H.A., Elfeil W.K., Nour A.A., Tantawy L., Kamel E.G., Eed E.M., El Askary A., Talaat S. (2021). Efficacy of the Newcastle Disease Virus Genotype VII.1.1-Matched Vaccines in Commercial Broilers. Vaccines.

[25] Steensels M., Soldan C., Rauw F., Roupie V., Lambrecht B. (2025). Protective efficacy of classical vaccines and vaccination protocols against an exotic Newcastle disease virus genotype VII.2 in Belgian layer and broiler chickens. Poult. Sci..

[26] de Graaf J.F., van Nieuwkoop S., de Meulder D., Lexmond P., Kuiken T., Groeneveld D., Fouchier R.A.M., van den Hoogen B.G. (2022). Assessment of the virulence for chickens of Newcastle Disease virus with an engineered multi-basic cleavage site in the fusion protein and disrupted V protein gene. Vet. Microbiol..

[27] Wang J.Y., Liu W.H., Ren J.J., Tang P., Wu N., Wu H.Y., Ching C.D., Liu H.J. (2015). Characterization of emerging Newcastle disease virus isolates in China. Virol. J..

[28] Liu J., Zhu J., Xu H., Li J., Hu Z., Hu S., Wang X., Liu X. (2017). Effects of the HN Antigenic Difference between the Vaccine Strain and the Challenge Strain of Newcastle Disease Virus on Virus Shedding and Transmission. Viruses.

[29] Dewidar A.A.A., Kilany W.H., El-Sawah A.A., Shany S.A.S., Dahshan A.-H.M., Hisham I., Elkady M.F., Ali A. (2022). Genotype VII.1.1-Based Newcastle Disease Virus Vaccines Afford Better Protection against Field Isolates in Commercial Broiler Chickens. Animals.

[30] Yang H.-M., Zhao J., Xue J., Yang Y.-L., Zhang G.-Z. (2017). Antigenic variation of LaSota and genotype VII Newcastle disease virus (NDV) and their efficacy against challenge with velogenic NDV. Vaccine.

[31] Ma S., Xia R., Wu W., Duan Z. (2025). Insights into the multifunctionality of viral glycoproteins F and HN in the lifecycle and pathogenesis of Newcastle disease virus: A systematic review. Vet. Res..

[32] Panda A., Huang Z., Elankumaran S., Rockemann D.D., Samal S.K. (2004). Role of fusion protein cleavage site in the virulence of Newcastle disease virus. Microb. Pathog..

[33] Iorio R.M., Field G.M., Sauvron J.M., Mirza A.M., Deng R., Mahon P.J., Langedijk J.P. (2001). Structural and functional relationship between the receptor recognition and neuraminidase activities of the Newcastle disease virus hemagglutinin-neuraminidase protein: Receptor recognition is dependent on neuraminidase activity. J. Virol..

[34] Porotto M., Salah Z., DeVito I., Talekar A., Palmer S.G., Xu R., Wilson I.A., Moscona A. (2012). The second receptor binding site of the globular head of the Newcastle disease virus hemagglutinin-neuraminidase activates the stalk of multiple paramyxovirus receptor binding proteins to trigger fusion. J. Virol..

[35] Cho S.H., Kwon H.J., Kim T.E., Kim J.H., Yoo H.S., Kim S.J. (2008). Variation of a newcastle disease virus hemagglutinin-neuraminidase linear epitope. J. Clin. Microbiol..

[36] Liu M., Shen X., Li J., Yu Y., Fan J., Jia X., Dai Y. (2023). Efficacy of Newcastle disease LaSota vaccine-induced hemagglutination inhibition antibodies against challenges with heterologous virulent strains of genotypes VII and IX. Vet. Immunol. Immunopathol..

[37] Tataje-Lavanda L., Málaga E., Verastegui M., Mayta Huatuco E., Icochea E., Fernández-Díaz M., Zimic M. (2023). Identification and evaluation in-vitro of conserved peptides with high affinity to MHC-I as potential protective epitopes for Newcastle disease virus vaccines. BMC Vet. Res..

[38] Jin Z., Wei Q., Bi Y., Li Y., Huo N., Mou S., Wang W., Liu H., Yang Z., Chen H. (2021). Identification of a potential neutralizing linear epitope of hemagglutinin-neuraminidase in Newcastle disease virus. Virol. J..

[39] Izquierdo-Lara R., Chumbe A., Calderón K., Fernández-Díaz M., Vakharia V.N. (2019). Genotype-matched Newcastle disease virus vaccine confers improved protection against genotype XII challenge: The importance of cytoplasmic tails in viral replication and vaccine design. PLoS ONE.

[40] Amoia C.F., Chengula A.A., Hakizimana J.N., Wambura P.N., Munir M., Misinzo G., Weger-Lucarelli J. (2024). Development of a genotype-matched Newcastle disease DNA vaccine candidate adjuvanted with IL-28b for the control of targeted velogenic strains of Newcastle disease virus in Africa. Vet. Res. Commun..

[41] Ren J., Chen Y., Yao R., Lai L., Yan X., Xiao H., Lin Q., Ren T., Chen L. (2025). Genotype-matched recombiant inactivated Newcastle disease virus vaccine confer protection against genotype XII challenge in geese with maternal antibodies. Poult. Sci..

[42] Park J.-K., Lee D.-H., Yuk S.-S., Tseren-Ochir E.-O., Kwon J.-H., Noh J.-Y., Kim B.-Y., Choi S.-W., Kang S.-M., Lee J.-B. (2014). Virus-Like Particle Vaccine Confers Protection against a Lethal Newcastle Disease Virus Challenge in Chickens and Allows a Strategy of Differentiating Infected from Vaccinated Animals. Clin. Vaccine Immunol..

[43] Firouzamandi M., Helan J.A., Moeini H., Soleimanian A., Khatemeh S., Hosseini S.D. (2023). Developing a vaccine against velogenic sub-genotype seven of Newcastle disease virus based on virus-like particles. AMB Express.

[44] Śmietanka K., Tyborowska J., Olszewska-Tomczyk M., Domańska-Blicharz K., Minta Z., Rabalski L., Czarnota A., Kucharczyk K., Szewczyk B. (2019). A Recombinant Turkey Herpesvirus Expressing F and HN Genes of Avian Avulavirus-1 (AAvV-1) Genotype VI Confers Cross-Protection against Challenge with Virulent AAvV-1 Genotypes IV and VII in Chickens. Viruses.

