# Supplementary material for: Newcastle Disease Virus Fusion and Haemagglutinin-Neuraminidase Gene Divergence: Implications for Vaccines
Source: Vet Sci. 2026 Apr 10;13(4):368. doi: 10.3390/vetsci13040368 (PMC13119920; doi:10.3390/vetsci13040368)
Supplement: Supplementary file 1 [file vetsci-13-00368-s001.zip › vetsci-4194316-supplementary.pdf]

Supplementary Table S1. Newcastle disease virus (NDV) fusion (F) and haemagglutinin-neuraminidase (HN) genes, analysed in the study

NDV-F gene sequences

S. No.	Accession	Host	Country	Year	Genotype	Pathotype
1	OR253543	Mallard	China	2020	I	Lentogenic
2	OR253551	Bar-headed goose	China	2017	I	Lentogenic
3	KT892755	Hooded crane	China	2015	I	Lentogenic
4	HM063422	Feral migratory ducks	China	2007	I	Lentogenic
5	AY562991	Chicken	Ireland	1967	I	Lentogenic
6	AY935500	Chicken	Australia	2006	I	Lentogenic
7	AY935499	Chicken	Australia	2005	I	Lentogenic
8	JX524203	Chicken	Australia	1966	I	Lentogenic
9	AY935492	Chicken	Australia	1998	I	Virulent
10	AY935493	Chicken	Australia	1998	I	Virulent
11	KP939089	Chicken	USA	1944	II	Virulent
12	JN863121	Chicken	USA	1948	II	Mesogenic
13	HQ902590	Quail	India	1998	II	Virulent
14	FJ939313	Chicken	Egypt	2005	II	Virulent
15	X04719	Chicken	USA	1945	II	Virulent
16	GU978777	Chicken	USA	1948	II	Virulent
17	AF077761	Chicken	USA	1946	II	Lentogenic
18	AY845400	Chicken	USA	1946	II	Lentogenic
19	PP788555	Chicken	Peru	2022	II	Virulent
20	OP169005	Chicken	USA	2021	II	Lentogenic
21	AF309418	Fowl	USA	1947	II	Lentogenic
22	KU133360	Black swan	Brazil	2009	II	Lentogenic
23	KU133356	Peregrine falcon	Brazil	2009	II	Virulent
24	JN872151	Chicken	USA	1947	II	Lentogenic
25	EU289028	Turkey	USA	1989	II	Lentogenic
26	JF950509	Chicken	China	2010	III	Mesogenic
27	OP751937	Chicken	China	2022	III	Virulent
28	FJ430159	Chicken	China	2005	III	Virulent
29	FJ430160	Goose	China	2005	III	Virulent
30	MZ306214	Chicken	China	2001	III	Virulent
31	MZ306216	Goose	China	2017	III	Virulent
32	EF201805	Chicken	China	2006	III	Mesogenic
33	MH996904	Pigeon	Bulgaria	1995	III	Mesogenic
34	GU182327	Chicken	Pakistan	1974	III	Virulent
35	MH092819	Chicken	Nigeria	1973	IV	Virulent
36	MK005971	Chicken	Bulgaria	1975	IV	Virulent
37	MH996952	Chicken	Nigeria	1973	IV	Virulent
38	MH996900	Pullet	Bulgaria	1959	IV	Virulent
39	KM056353	Chicken	India	2014	IV	Virulent
40	AY562990	Psittacine	USA	1971	V	Virulent
41	JQ247691	Chicken	USA	1971	V	Virulent
42	EU518682	Dove	Mexico	2004	V	Virulent
43	KJ577136	Chicken	Mexico	1973	V	Virulent
44	KC808510	Scarlet macaw	Mexico	2009	V	Virulent
45	EU518684	Chicken	Mexico	2006	V	Virulent

46	HM117720	Chicken	Mexico	2005	V	Virulent
47	EU518683	Chicken	Mexico	2005	V	Virulent
48	EU477192	Eurasian collared dove	USA	2005	VI	Virulent
49	EU477188	Dove	USA	2004	VI	Virulent
50	EU477190	Pigeon	USA	2004	VI	Virulent
51	EU477189	Pigeon	USA	2000	VI	Virulent
52	KU059752	Eurasian collared dove	USA	2004	VI	Virulent
53	KP780870	Rock pigeon	USA	2013	VI	Virulent
54	JQ319052	Chicken	Venezuela	2008	VII	Virulent
55	MT668587	Chicken	China	2008	VII	Virulent
56	MT091963	Chicken	Botswana	2019	VII	Virulent
57	OP378144	Chicken	Bangladesh	2020	VII	Virulent
58	MT876630	Chicken	Turkey	2016	VII	Virulent
59	MT424733	Chicken	Macedonia	2020	VII	Virulent
60	MH715892	Game fowl	China	2011	VIII	Virulent
61	FJ751919	Chicken	China	1985	VIII	Virulent
62	MG456905	Chicken	China	1946	IX	Virulent
63	KC424431	White-cheeked starling	China	2008	IX	Virulent
64	KC424428	Whooper swan	China	2008	IX	Virulent
65	KR014814	Duck	China	2014	IX	Virulent
66	MT668582	Black swan	China	2019	IX	Virulent
67	KX857723	Blue-winged teal	USA	2008	X	Lentogenic
68	KX857720	Mallard	USA	2010	X	Lentogenic
69	EF564832	Mallard	USA	1986	X	Lentogenic
70	EF564826	Northern pintail	USA	1987	X	Lentogenic
71	MK006020	Turkey	USA	2009	X	Lentogenic
72	JX518884	Chicken	Madagascar	2011	XI	Virulent
73	JX518881	Chicken	Madagascar	2010	XI	Virulent
74	JX518877	Chicken	Madagascar	2011	XI	Virulent
75	HQ266602	Chicken	Madagascar	2008	XI	Virulent
76	JN627508	Goose	China	2011	XII	Virulent
77	KR732614	Peacock	Peru	2011	XII	Virulent
78	JN800306	Chicken	Peru	2008	XII	Virulent
79	OP066060	Chicken	Colombia	2009	XII	Virulent
80	MK934289	Chicken	Bangladesh	2010	XIII	Virulent
81	PQ333012	Chicken	Bangladesh	2023	XIII	Virulent
82	GU182331	Chicken	Pakistan	2007	XIII	Virulent
83	MT362714	Chicken	India	2018	XIII	Virulent
84	MH996908	Chicken	Nigeria	2009	XIV	Virulent
85	KC568209	Chicken	Nigeria	2009	XIV	Virulent
86	KC568206	Helmeted guineafowl	Nigeria	2009	XIV	Virulent
87	MT543162	Chicken	Niger	2019	XIV	Virulent
88	MT543161	Chicken	Niger	2019	XIV	Virulent
89	JX915243	Chicken	Mexico	1947	XVI	Virulent
90	JX915242	Chicken	Dominican Republic	1986	XVI	Virulent
91	JX186997	Chicken	Dominican Republic	2008	XVI	Virulent
92	JX119193	Chicken	Dominican Republic	2008	XVI	Virulent

93	MH392226	Chicken	Dominican Republic	2008	XVI	Virulent
94	HF969185	Chicken	Ivory Coast	2007	XVII	Virulent
95	FJ772458	Chicken	Burkina Faso	2008	XVII	Virulent
96	HF969188	Chicken	Nigeria	2008	XVII	Virulent
97	HF969194	Chicken	Nigeria	2009	XVII	Virulent
98	HF969196	Chicken	Nigeria	2009	XVII	Virulent
99	MT543158	Chicken	Niger	2019	XVIII	Virulent
100	JX390609	Chicken	Togo	2009	XVIII	Virulent
101	FJ772466	Chicken	Ivory Coast	2008	XVIII	Virulent
102	HF969218	Chicken	Ivory Coast	2007	XVIII	Virulent
103	HF969126	Duck	Ivory Coast	2006	XVIII	Virulent
104	GU332662	Cormorant	USA	2008	XIX	Virulent
105	GU332655	Cormorant	USA	2008	XIX	Virulent
106	JN255782	Double-crested cormorant	USA	2010	XIX	Virulent
107	JN255779	European herring gull	USA	2010	XIX	Virulent
108	EF520718	Game fowl	USA	2002	XIX	Virulent
109	EU518677	Chicken	Mexico	2000	XIX	Virulent
110	AY288993	Chicken	Honduras	2000	XIX	Virulent
111	MZ101335	Chicken	Ukraine	2003	XX	Virulent
112	KY042125	Chicken	Bulgaria	1992	XX	Virulent
113	KY042142	Quail	South Korea	1988	XX	Virulent
114	KY042143	Chicken	South Korea	1993	XX	Virulent
115	MZ101333	Chicken	Ukraine	1985	XX	Virulent
116	KU862299	Pigeon	Pakistan	2015	XXI	Virulent
117	OP641847	Pigeon	Bangladesh	2020	XXI	Virulent
118	MH996953	Pigeon	Nigeria	2007	XXI	Virulent
119	KY042134	Pigeon	Egypt	2015	XXI	Virulent
120	KJ914672	Pigeon	Ukraine	2011	XXI	Virulent
121	MZ101341	Pigeon	Ukraine	2014	XXI	Virulent

NDV-HN gene sequences

S. No.	Accession	Host	Country	Year	Genotype	Pathotype
1	AY935493	Chicken	Australia	1998	I	Virulent
2	AY935492	Chicken	Australia	1998	I	Virulent
3	JX524203	Chicken	Australia	1966	I	Lentogenic
4	AY935500	Chicken	Australia	2006	I	Lentogenic
5	AY935499	Chicken	Australia	2005	I	Lentogenic
6	HM063422	Feral migratory ducks	China	2007	I	Lentogenic
7	AY562991	Chicken	Ireland	1967	I	Lentogenic
8	HQ902590	Quail	India	1998	II	Virulent
9	FJ939313	Chicken	Egypt	2005	II	Virulent
10	GU978777	Chicken	USA	1948	II	Virulent
11	AF077761	Chicken	USA	1946	II	Lentogenic
12	AY845400	Chicken	USA	1946	II	Lentogenic
13	PP788555	Chicken	Peru	2022	II	Virulent
14	OP169005	Chicken	USA	2021	II	Lentogenic
15	AF309418	Fowl	USA	1947	II	Lentogenic
16	EU289028	Turkey	USA	1989	II	Lentogenic

17	JF950509	Chicken	China	2010	III	Mesogenic
18	OP751937	Chicken	China	2022	III	Virulent
19	FJ430159	Chicken	China	2005	III	Virulent
20	FJ430160	Goose	China	2005	III	Virulent
21	MZ306214	Chicken	China	2001	III	Virulent
22	MZ306216	Goose	China	2017	III	Virulent
23	EF201805	Chicken	China	2006	III	Mesogenic
24	MH996952	Chicken	Nigeria	1973	IV	Virulent
25	MH996900	Pullet	Bulgaria	1959	IV	Virulent
26	KM056353	Chicken	India	2014	IV	Virulent
27	MH996899	Chicken	Nigeria	1973	IV	Virulent
28	AY562990	Psittacine	USA	1971	V	Virulent
29	JQ247691	Chicken	USA	1971	V	Virulent
30	HM117720	Chicken	Mexico	2005	V	Virulent
31	KJ577136	Chicken	Mexico	1973	V	Virulent
32	KU059752	Eurasian collared dove	USA	2004	VI	Virulent
33	MZ306218	Peacock	China	2001	VI	Virulent
34	MZ458602	Pigeon	Brazil	2019	VI	Virulent
35	MZ306222	Turtle dove	China	2018	VI	Virulent
36	MK046921	Pigeon	Mexico	2017	VI	Virulent
37	MN557411	Chicken	Indonesia	2014	VII	Virulent
38	MH371037	Chicken	Israel	2011	VII	Virulent
39	KR676419	Chicken	Pakistan	2015	VII	Virulent
40	KM408752	Goose	China	2010	VII	Virulent
41	MK673139	Chicken	Egypt	2011	VII	Virulent
42	MT370497	Chicken	Iraq	2004	VII	Virulent
43	MG867723	Chicken	China	2008	VII	Virulent
44	MH715892	Game fowl	China	2011	VIII	Virulent
45	FJ751919	Chicken	China	1985	VIII	Virulent
46	MF285077	Chicken	China	1998	VIII	Virulent
47	MG456905	Chicken	China	1946	IX	Virulent
48	KR014814	Duck	China	2014	IX	Virulent
49	KC424433	Whooper swan	China	2008	IX	Virulent
50	KC424434	Blackbird	China	2008	IX	Virulent
51	KC424436	White-cheeked starling	China	2008	IX	Virulent
52	KF219497	Duck	China	2010	IX	Virulent
53	HQ266602	Chicken	Madagascar	2008	XI	Virulent
54	HQ266603	Chicken	Madagascar	1992	XI	Virulent
55	HQ266604	Chicken	Madagascar	2008	XI	Virulent
56	KR732614	Peacock	Peru	2011	XII	Virulent
57	JN800306	Chicken	Peru	2008	XII	Virulent
58	OP066060	Chicken	Colombia	2009	XII	Virulent
59	KC152048	Goose	China	2011	XII	Virulent
60	PP788551	Chicken	Peru	2005	XII	Virulent
61	MT409244	Chicken	India	2015	XIII	Virulent
62	OR230623	Chicken	Tanzania	2018	XIII	Virulent
63	MK796809	Chicken	India	2016	XIII	Virulent
64	MF422128	Chicken	India	2013	XIII	Virulent
65	KY171994	Chicken	Nigeria	2009	XIV	Virulent
66	MH996942	Mallard	Nigeria	2009	XIV	Virulent
67	MH996943	Pigeon	Nigeria	2009	XIV	Virulent

68	MH996954	Chicken	Nigeria	2010	XIV	Virulent
69	JX119193	Chicken	Dominican Republic	2008	XVI	Virulent
70	HF969185	Chicken	Ivory Coast	2007	XVII	Virulent
71	HF969188	Chicken	Nigeria	2008	XVII	Virulent
72	HF969194	Chicken	Nigeria	2009	XVII	Virulent
73	HF969196	Chicken	Nigeria	2009	XVII	Virulent
74	JX390609	Chicken	Togo	2009	XVIII	Virulent
75	MH392227	Chicken	Nigeria	2009	XVIII	Virulent
76	HF969218	Chicken	Ivory Coast	2007	XVIII	Virulent
77	AY288993	Chicken	Honduras	2000	XIX	Virulent
78	MK673141	Double-crested cormorant	USA	2010	XIX	Virulent
79	KY042125	Chicken	Bulgaria	1992	XX	Virulent
80	MH996953	Pigeon	Nigeria	2007	XXI	Virulent
81	KY042134	Pigeon	Egypt	2015	XXI	Virulent
